# Analysis of the characteristics and risk factors affecting the judgment results of medical damage liability disputes in 3172 second-instance and retrial cases in China

**DOI:** 10.1186/s12960-023-00832-6

**Published:** 2023-06-29

**Authors:** Yanfei Shen, Sheng Lei, Qi Wang, Hongjing Wang, Xiangyong Hao, Hui Cai

**Affiliations:** 1 School of Marxism, Lanzhou University, Lanzhou, China; 2 Department of Medical Management, Gansu Provincial Hospital, Lanzhou, China; 3 Ray Vow of Gansu Law Firm, Lanzhou, China; 4 Department of General Surgery, Gansu Provincial Hospital, Lanzhou, China

**Keywords:** Medical damage liability disputes, Second-instance, Retrial cases, Compensation, China

## Abstract

**Background:**

Medical disputes remain a global public health problem. However, an analysis of the characteristics and risk factors affecting the judgment results of medical damage liability disputes in second-instance and retrial cases in China has yet to be conducted.

**Methods:**

We conducted a systematic search and evaluation of second-instance and retrial cases among all medical damage liability disputes in China Judgments Online; SPSS 22.0 was used for the statistical analysis. A *χ*^2^ test or likelihood ratio Chi-square test was used to compare differences between groups, and multivariate logistic regression analysis was performed to determine independent risk factors that could affect the judgment results of medical disputes.

**Results:**

We included 3172 second-instance and retrial cases among all medical damage liability disputes in the analysis. The results showed that 48.04% of cases were unilateral appeals by the patient, and medical institutions were responsible for providing compensation in 80.64% of these cases. Cases involving compensation ranged from Chinese Yuan (CNY) 100 000 to 500 000 ranked first (40.95%); 21.66% were non-compensation cases. Cases involving mental damage compensation of less than CNY 20 000 accounted for 39.03%. Violations of medical treatment and nursing routines accounted for 64.25% of all cases. In addition, re-identification in 54.59% of cases changed the initial appraisal opinion. Independent risk factors for medical personnel to lose a lawsuit in a multivariate logistic regression model included appeal originator [patient side: OR = 18.809 (95% CI 11.854–29.845); both sides: OR = 22.168 (95% CI 12.249–40.117)], change of the original verdict (OR = 5.936, 95% CI 3.875–9.095), judicial identification (OR = 6.395, 95% CI 4.818–8.487), violations of medical treatment and nursing routines (OR = 8.783, 95% CI 6.658–11.588), and non-standard medical document writing (OR = 8.500, 95% CI 4.805–15.037).

**Conclusion:**

Our study clarifies the characteristics of second-instance and retrial cases among all medical damage liability disputes in China from multiple perspectives and identifies the independent risk factors for medical personnel losing a lawsuit. This study could help medical institutions prevent and reduce medical disputes, at the same time, it could be helpful for medical institutions to provide better medical treatment and nursing services for patients.

**Supplementary Information:**

The online version contains supplementary material available at 10.1186/s12960-023-00832-6.

## Introduction

Healthcare development is closely related to citizens’ quality of life. However, medical disputes remain a global public health problem [1–3]. According to the World Health Organization, approximately 8–38% of healthcare workers worldwide were subject to medical disputes in 2019 [4]. This figure is even higher in Asia [5, 6].

Medical disputes seriously negatively impact healthcare workers’ physical and mental health, leading to anxiety, depression, job burnout, and job dissatisfaction [7–17]. Furthermore, disputes are directly or indirectly negatively correlated with professional efficacy and seriously affect the doctor–patient relationship, increasing the incidence of defensive medical practice among doctors and eventually reducing the quality of health services [13, 18–20]. Therefore, long-term medical disputes are likely to have a far-reaching negative impact on medical and healthcare systems. The report of a 2021 physician survey published by the department of Social Sciences of Tsinghua University revealed that 57.95% of doctors report being afraid or very afraid of being the subject of a dispute [21, 22].

Conversely, medical disputes also correspondingly promote medical managers to find problems in the medical process and pay more attention to strengthen medical staff’s training in normalized diagnoses and treatment, communication skills, humanistic medical education which improving healthcare workers’ humanistic medical literacy [10, 23–27]. Moreover, medical disputes stimulated medical personnel to attach great importance to health education for patients and their families, which will make them have a clearer and reasonable understanding of their diseases and will be able to more easily accept the adverse effects of such diseases [10]. In view of this, it is of great practical importance to study how to help medical personnel improve doctor–patient relationships and prevent medical disputes.

Overviews of medical disputes and violence against health professionals and facilities in China are available [10, 28–30]. However, no studies have been conducted to systematically explore the characteristics, judicial identification procedures, and risk factors of complex medical disputes, such as second-instance and retrial cases, from a national perspective. Therefore, we conducted a multi-dimensional analysis of the judicial results of second-instance and retrial cases in China, aiming to understand the characteristics and influencing factors of such cases, help medical personnel improve awareness and prevent medical disputes, and provide a basis for relevant departments to formulate scientific and reasonable strategies to reduce medical disputes.

## Materials and methods

### Inclusion and exclusion criteria

The inclusion criteria were as follows: second-instance and retrial cases among all medical damage liability disputes in China Judgments Online (https://wenshu.court.gov.cn) from January 1, 2018, to December 31, 2018. No restrictions were placed regarding case origin or the age, sex, and ethnicity of the patients. Cases that did not lead to a complete judgment were excluded.

### Case retrieval

Two researchers independently used the keywords “medical damage liability disputes”, “medical disputes”, “doctor–patient disputes”, “second instance”, and “retrial instance” for systematic retrieval. When the information provided in the case judgment was incomplete, we contacted the publisher to verify additional information and determine the retrieved case outcomes. The two researchers (YS and SL) independently read the titles and abstracts of all the retrieved cases one by one, excluded cases that did not meet the inclusion criteria, and independently read the full text of each remaining case to determine whether they truly fulfilled the inclusion and exclusion criteria. All the researchers were hospital managers with substantial practical experience in medical dispute mediation or practicing lawyers with extensive experience in medical damage compensation liability disputes.

### Data extraction

According to the self-designed data extraction form, the two researchers independently collected and cross-checked the extracted data from the second-instance and retrial cases that met the inclusion and exclusion criteria. The contents of the extracted data are listed in the following table. Among these variables, judicial identification status refers to the final legal opinion made by the court after the case is brought to the court and the court entrusts the professional appraisal institution to issue professional opinions on the diagnosis and treatment according to the authority of the court or the application of the parties. The court will determine the amount of compensation borne by the parties according to the proportion of responsibility made by judicial appraisal. Violations of medical treatment and nursing routines refers to the violation of laws and administrative regulations issued by the government, such as the “Civil Code”, the “Physician Law”, and the “Nurse Management Regulations”, and violation of various professional diagnosis and treatment norms, diagnosis and treatment guidelines and expert consensus.

### Statistical analysis

Excel 2016 software was used to collate data. The database was revisited in cases with missing information on individual variables, or such information was filled in according to the majority to ensure data integrity. Frequency statistics were used to indicate the proportion of events. GraphPad Prism 7 (GraphPad Software) was used for mapping analyses.

To further explore the correlation between the judgment results of the included second-instance and retrial cases and the relevant variables, the judgment result was used as the outcome variable, and the Chi-square test was used to compare the differences between cases in which the medical side lost the lawsuit and those in which this side won the lawsuit based on the relevant variables. A *χ*^2^ test or likelihood ratio Chi-square test was used to compare differences between groups. Taking judgment results as the dependent variable, the medical-side loss of a lawsuit as the positive event was assigned a value of “1” and the medical-side win a lawsuit as the negative event was assigned a value of “0”. The independent variables are listed in the following supplementary table based on the variable type. SPSS 22.0 software was used for the statistical analysis. Multivariate logistic regression analysis was performed on the factors that may affect the judgment results of medical disputes. *P* < 0.05 was considered to indicate statistical significance.

## Results

**Fig. 1:**
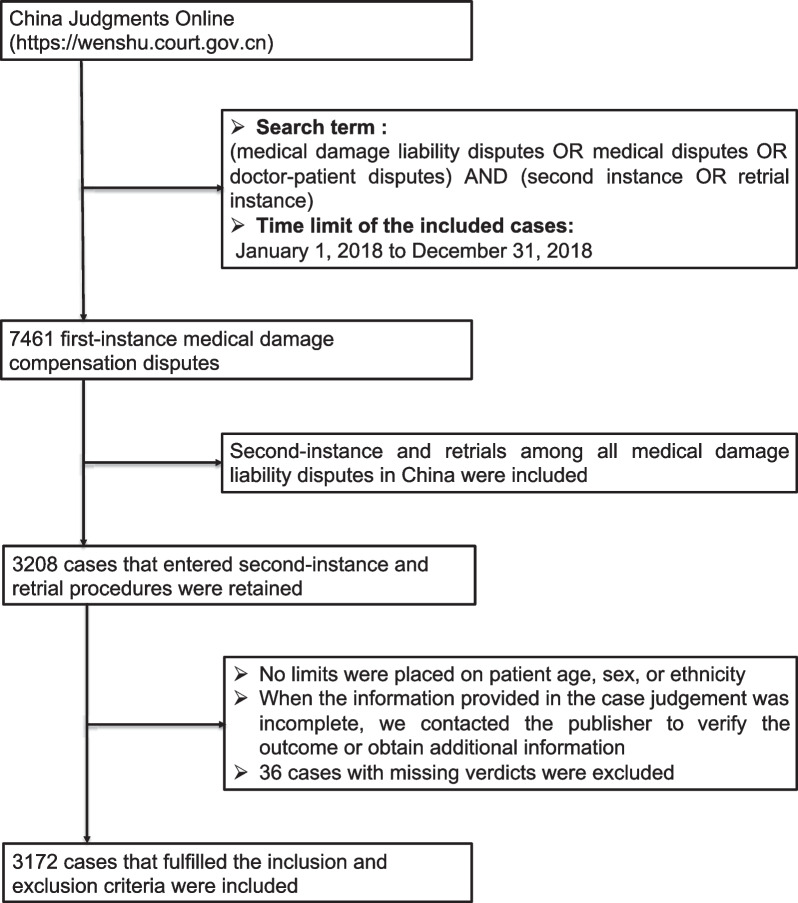
Flowchart depicting case screening

From January 1, 2018, to December 31, 2018, the national courts judged a total of 7461 first-instance medical damage compensation disputes reported in China Judgments Online; 3208 of these cases entered second-instance and retrial procedures, with an appeal rate of 43%. According to the inclusion and exclusion criteria, after 36 cases with missing verdicts were excluded, 3172 second-instance and retrial medical damage liability disputes were included in the analysis. The flowchart of case screening is shown in Fig. 1.

### Overall situation of the included second-instance and retrial cases

**Fig. 2:**
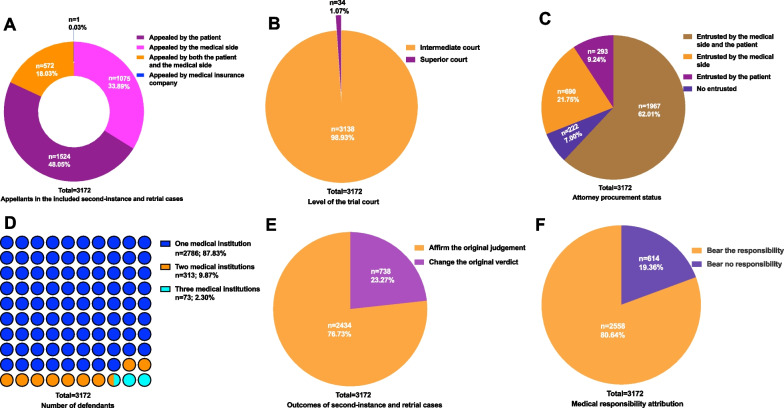
Overview of second-instance and retrial cases. **A** Appellants in the included second-instance and retrial cases; **B** level of the trial court; **C** attorney procurement status; **D** number of defendants; **E** outcomes of second-instance and retrial cases; **F** medical responsibility attribution

As shown in Fig. 2A, classification based on appeal originator revealed that 48.05% of the second-instance and retrial cases were unilaterally appealed by the patient, 33.89% by the medical side, and 18.03% by both parties. By analyzing the types of appeal originators, we can infer that if the appeal changes an earlier decision, it is likely that the change will favor the patients. The Intermediate Court is responsible for cases of second-instance appealed by the subordinate grassroots courts. The High Court is responsible for cases of first-instance with significant influence in the province and the second-instance and retrial cases appealed by the subordinate municipal Intermediate Court. As shown in Fig. 2B, 98.93% of second-instance and retrial cases were tried in Intermediate Courts, and 1.07% were tried by the Superior Court.

Regarding the situation of procuration by the attorney, as shown in Fig. 2C, 62.01% of second-instance and retrial cases involving both medical and patient appellants were entrusted to a lawyer agency; this proportion was 21.75% among medical appellants alone and 9.24% among patients alone. It can be seen from this result that most patients tend to choose a professional lawyer teams to safeguard their own rights and interests when facing necessary medical dispute litigation cases, which also indicates that patients’ awareness of the rule of law is constantly improving. A total of 222 cases (7%) were not entrusted to a lawyer agency. In addition, the number of defendants is shown in Fig. 2D; one medical institution was the defendant in 87.83% of cases, two in ~ 10% of cases, and three in 2.30% of cases.

The second-instance and retrial case verdicts are shown in Fig. 2E shows; in 76.73% of cases, the original judgment was affirmed, while in 23.27%, the verdict changed. It shows that the majority of the second-instance and retrial case verdicts maintain the first trial judgment results, the responsibility of the judgment subject is clear. As shown in Fig. 2F, 80.64% of second-instance and retrial cases resulted in medical institutions bearing the responsibility for providing compensation; in 19.36% of cases, the medical institution bore no responsibility.

### Case compensation in the included second-instance and retrial cases

**Fig. 3:**
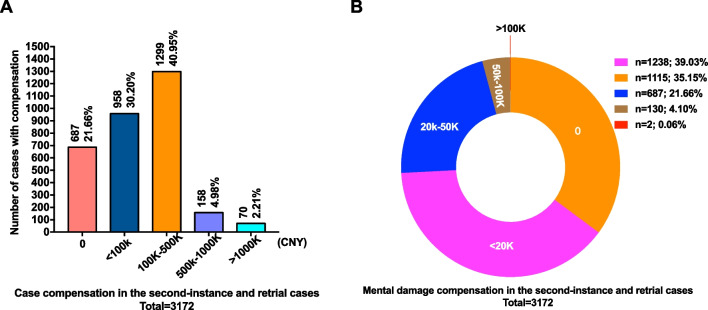
Case compensation in included second-instance and retrial cases. **A** Case compensation in the second-instance and retrial cases; **B** mental damage compensation in the second-instance and retrial cases

As shown in Fig. 3A, the majority of cases involved compensation ranged from Chinese Yuan (CNY) 100 000 to 500 000 (40.95%). In 30.2% of cases, the amount of compensation was under CNY 100 000. Non-compensation cases accounted for 21.66%. Compensation between CNY 500 000 and 1 000 000 was awarded in 4.98% of cases. Finally, in 2.21% of cases, compensation exceeded CNY 1 000 000.

As shown in Fig. 3B, in 39.03% of cases, the amount of mental damage compensation was less than CNY 20 000; in 21.66%, compensation ranged from CNY 20 000 to 50 000. In 4.10% of cases, mental damage compensation ranged from CNY 50 000 to 100 000, and 0.06% were awarded over CNY 100 000. In 35.15% of the cases, medical institutions were not responsible for providing compensation for mental damage. Such results also remind medical institutions that once the result of medical litigation is lost, in addition to bear the compensation for patients losses directly caused by the responsibility of medical institutions, it is inevitable to bear the compensation for patients mental losses. If such cases are more, there may be adverse effects on the development of medical institutions.

### Medical factors in the included second-instance and retrial cases

**Fig. 4:**
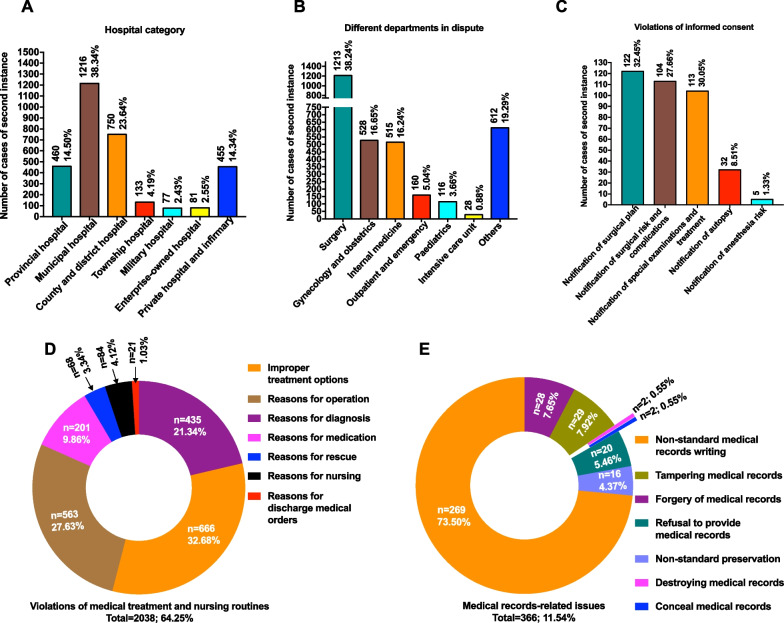
Medical factors in the included second-instance and retrial cases. **A** Hospital category; **B** departments in dispute; **C** violations of informed consent; **D** violations of medical treatment and nursing routines; **E** medical records-related issues

As shown in the statistics by hospital category in Fig. 4A, municipal hospitals experience the highest number of medical damage liability disputes, accounting for 38.34% of all cases, followed by county and district hospitals, accounting for 23.64%. As shown in Fig. 4B, regarding medical damage liability disputes by department, surgery departments are subject to the highest number of disputes (38.24%), followed by obstetrics and gynecology (16.65%) and internal medicine (16.24%).

The common causes of medical damage liability disputes were subdivided as follows. Violation of informed consent is shown in Fig. 4C; the proportion of violation of the notification of surgical plan was the highest, reaching 32.45%, followed by 30.05% for violation of the notification of special examination and treatment, and 27.66% for violation of the notification of surgical risks and complications. Violation of the notification of autopsy accounted for 8.51%. Violation of the notification of anesthesia accounted for 1.33%.

Violations of medical treatment and nursing routines are shown in Fig. 4D; these violations accounted for 64.25% of all cases and 79.67% of all cases of responsibility. Among them, improper treatment options accounted for 32.68%, incorrect causes of surgery accounted for 27.63%, improper diagnosis accounted for 21.34%, improper medication accounted for 9.86%, violations of nursing routines accounted for 4.12%, improper rescue accounted for 3.34%, and improper discharge medical orders accounted for 1.03%.

Medical records-related issues are shown in Fig. 4E; the cases with responsibility due to non-standard medical records writing accounted for 73.50% of all compensation cases, followed by cases of tampering and forgery, accounting for 7.92% and 7.65%, respectively. Cases with responsibility due to refusal to provide medical records accounted for 5.46%, and those involving non-standard medical records storage accounted for 4.37%. There were two cases of destruction and concealment of medical records, accounting for 0.55%.

### Related factors affecting the judgment results of the included second-instance and retrial cases in cases judicial identification procedure

**Fig. 5:**
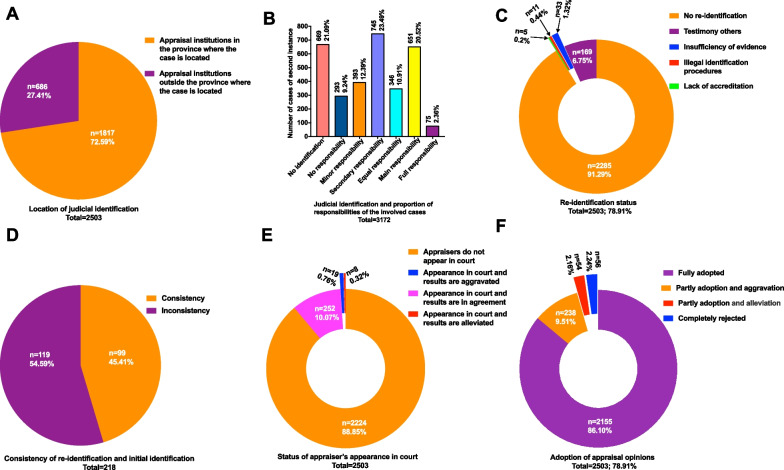
Related factors affecting the judgment results of the included second-instance and retrial cases based on judicial identification procedure. **A** Location of judicial identification; **B** judicial identification and proportion of responsibilities of the involved cases; **C** re-identification status; **D** comparison of re-identification and initial judicial identification; **E** status of appraiser’s appearance in court; **F** adoption of appraisal opinions

As shown in Fig. 5A, 72.58% of the second-instance and retrial cases were identified in the appraisal institutions in the province where the case is located, and 27.42% were identified in appraisal institutions outside of the province. According to the judicial identification results in Fig. 5B, the proportion of secondary responsibility and main responsibility of the medical side in the included second-instance and retrial cases is higher (23.49% and 20.52%, respectively). Full responsibility was taken in 2.36% of the included cases, and no responsibility was taken in 9.24%. In addition, there was no application to the court for identification in 21.09% of the included cases.

In total, 2503 cases were considered for possible re-identification. Re-identification data are shown in Fig. 5C; 91.29% of the included cases failed to undergo re-identification, indicating that the initial appraisal opinion is crucial. In addition, 1.32%, 0.44%, and 0.2% of the included cases underwent re-identification due to insufficient evidence, illegal identification procedures, and lack of accreditation, respectively. Moreover, 6.75% of the included cases involved the testimony of others. The comparison of re-identification and initial judicial identification of the involved cases is shown in Fig. 5D; in 54.59% of cases, re-identification changed the initial appraisal opinion, and the decision did not change in 45.41%.

Whether the appraiser appeared in court in the involved cases is shown in Fig. 5E; the proportion of cases in which the appraisers did not appear in court was 88.85%. The proportion of cases resulting in decision agreement in which the appraisers appeared in court was 10.07%, the appraisers appear in court and the results are aggravated in the end accounted for 0.76%, and the appraisers appear in court and the results are alleviated in the end accounted for 0.32%.

As shown in Fig. 5F, the proportion of cases in which the court fully adopted the appraisal opinions put forth by institutions accounted for 86.10%. The proportion of cases in which the court partly adopted the appraisal opinions and increased the proportion of responsibility accounted for 9.51%. The proportion of responsibility was reduced in 2.16% of cases with partially adopted appraisal opinions, and in 2.24% of cases, the court completely rejected the appraisal opinions.

### Correlation analysis between judgment results of the included second-instance and retrial cases and the related factors

The variable assignment table involved in the analysis is shown in Additional file 1: Table S1. As shown in Table 1, except for the three variables of the level of the trial court, hospital category, and re-identification status, the distribution of the remaining variables has a statistically significant difference when the medical side lost a lawsuit vs. when this side won a lawsuit (all *P* < 0.05).

**Table 1:** Correlation analysis between judgment results of the included second-instance and retrial cases and the related factors

Variables	Total	Constituent ratio	Medical side wins (*N* (%))	Medical side loses (*N* (%))	*χ*^2^	*P*
Appeal originator					631.944	< 0.001
Medical side	1075	33.89%	23 (3.79)	1052 (41.01)		
Both parties	572	18.03%	14 (2.31)	558 (21.75)		
Patient side	1524	48.05%	570 (93.90)	955 (37.23)		
Medical insurance company	1	0.03%				
Total	3172		607	2565		
Level of the trial court					2.345	0.126
Intermediate court	3138	98.93%	597 (98.35)	2541 (99.06)		
Superior court	34	1.07%	10 (1.65)	24 (0.94)		
Total	3172		607	2565		
Procurement of attorney					76.769	< 0.001
Without entrusted	222	7.00%	69 (11.37)	153 (5.96)		
Entrusted by the medical side	690	21.75%	186 (30.64)	504 (19.65)		
Entrusted by the patient side	293	9.24%	66 (10.87)	227 (8.85)		
Entrusted by both parties	1967	62.01%	286 (47.12)	1681 (65.64)		
Total	3172		607	2549		
Outcomes of second-instance and retrial cases					136.142	< 0.001
Affirmed the original judgement	2434	77.12%	575 (94.72)	1859 (72.48)		
Changed the original verdict	738	23.38%	32 (5.27)	706 (27.52)		
Total	3172		607	2565		
Hospital category					3.013	0.807
Township hospital	133	4.19%	24 (3.95)	109 (4.24)		
County and district hospital	750	23.64%	143 (23.56)	607 (23.66)		
Municipal hospital	1216	38.34%	218 (35.91)	998 (38.91)		
Private hospital and infirmary	455	14.34%	94 (15.49)	361 (14.07)		
Enterprise-owned hospital	81	2.55%	17 (2.80)	64 (2.50)		
Military hospital	77	2.43%	15 (2.47)	62 (2.43)		
Provincial hospital	460	14.50%	96 (15.82)	364 (14.19)		
Total	3172		607	2565		
Number of defendants					6.293	0.043
One medical institution	2786	87.83%	544 (89.62)	2242 (87.41)		
Two medical institutions	313	9.87%	45 (7.41)	268 (10.45)		
Three medical institutions	73	2.30%	18 (2.97)	55 (2.14)		
Total	3172		607	2565		
Departments in dispute					58.160	< 0.001
Outpatient and emergency	160	5.04%	36 (5.93)	124 (4.83)		
Pediatrics	116	3.66%	22 (3.62)	94 (3.66)		
Obstetrics and gynecology	528	16.65%	61 (10.05)	467 (18.21)		
Intensive care unit	28	0.88%	6 (0.99)	22 (0.86)		
Internal medicine	515	16.24%	104 (17.13)	411 (16.02)		
Surgery	1213	38.24%	205 (33.77)	1008 (39.30)		
Other departments	612	19.29%	173 (28.50)	439 (17.12)		
Total	3172		607	2565		
Judicial identification status and proportion of responsibilities					1313.540	< 0.001
No identification	669	21.09%	362 (59.64)	307 (11.97)		
No responsibility	293	9.24%	189 (31.14)	104 (4.05)		
Minor responsibility	393	12.39%	9 (1.48)	384 (14.97)		
Secondary responsibility	745	23.49%	28 (4.61)	717 (27.95)		
Equal responsibility	346	10.91%	6 (0.99)	340 (13.25)		
Main responsibility	651	20.52%	13 (2.14)	638 (24.87)		
Full responsibility	75	2.36%	0 (0.00)	75 (2.92)		
Total	3172		607	2565		
Adoption of appraisal opinions					696.165	< 0.001
Fully adopted	2155	86.10%	223 (91.02)	1932 (85.56)		
Partially adopted and aggravation	238	9.51%	2 (0.82)	236 (10.45)		
Partially adopted and alleviation	54	2.16%	4 (1.63)	50 (2.21)		
Completely rejected	56	2.24%	16 (6.53)	40 (1.77)		
Total	2503		245	2258		
Location of judicial identification					48.429	< 0.001
Appraisal institutions in the province	1817	72.59%	224 (91.43)	1593 (70.55)		
Appraisal institutions outside the province	686	27.41%	21 (8.57)	665 (29.45)		
Total	2503		245	2258		
Re-identification status					3.729	0.513
No re-identification	2285	91.29%	303 (89.91)	1982 (91.51)		
Illegal identification procedures	11	0.44%	1 (0.30)	10 (0.46)		
Insufficiency of evidence	33	1.32%	7 (2.07)	26 (1.20)		
Testimony others	169	6.75%	26 (7.72)	143 (6.60)		
Lack of accreditation	5	0.20%	0	5 (0.23)		
Total	2503		337	2166		
Comparison of re-identification and initial judicial identification					11.368	0.010
Consistency	99	45.41%	22 (68.75)	77 (41.40)		
Inconsistency	119	54.59%	10 (31.25)	109 (58.60)		
Total	218		32	186		
Appraiser appearance in court					64.692	< 0.001*
Appraisers do not appear in court	2224	88.85%	371 (90.49)	1853 (88.53)		
Appear in court and the results are in agreements	252	10.07%	26 (6.34)	216 (10.32)		
Appear in court and the results are aggravated	19	0.76%	0 (0.00)	19 (0.91)		
Appear in court and the results are alleviated	8	0.32%	3 (0.73)	5 (0.24)		
Total	2503		410	2093		
Amount of compensation (CNY)					2184.060	< 0.001
No compensation	687	21.66%	554 (91.27)	133 (5.19)		
0–100 000	958	30.20%	16 (2.64)	942 (36.73)		
100 000–500 000	1299	40.95%	11 (1.81)	1288 (50.21)		
500 000–1 000 000	158	4.98%	4 (0.66)	154 (6.00)		
More than 1 000 000	70	2.21%	22 (3.62)	48 (1.87)		
Total	3172		607	2565		
Mental damage compensation (CNY)					1227.876	< 0.001*
No compensation	1115	35.15%	584 (96.21)	531 (20.70)		
0–20 000	1238	39.03%	11 (1.81)	1227 (47.84)		
20 000–50 000	687	21.66%	11 (1.81)	676 (26.35)		
50 000–100 000	130	4.10%	1 (0.16)	129 (5.03)		
More than 100 000	2	0.06%	0 (0.00)	2 (0.07)		
Total	3172		607	2565		
Informed consent notification					42.816	< 0.001
Not violated	2796	88.15%	591 (97.36)	2205 (85.96)		
Violated	376	11.85%	16 (2.64)	360 (14.04)		
Total	3172		607	2565		
Medical records					57.402	< 0.001
Standard	2806	88.46%	590 (97.20)	2216 (86.39)		
Non-standard	366	11.54%	17 (2.80)	349 (13.61)		
Total	3172		607	2565		
Violations of medical treatment and nursing routines					740.597	< 0.001
Not violated	1134	35.75%	505 (83.20)	629 (24.52)		
Violated	2038	64.25%	102 (16.80)	1936 (75.48)		
Total	3172		607	2565		

*Likelihood ratio

Additionally, other important information is shown in Table 1. Regarding judicial identification and proportion of responsibilities, no judicial identification (59.64%) may be an adverse factor leading to the patient-side loss of the lawsuit. In terms of compensation, nearly 94.81% of the included cases required compensation when the medical side lost a lawsuit, which ranged from CNY 100 000 to 500 000 in 50.21% of the cases. Nearly 79.30% of the included cases required mental damage compensation, which was less than CNY 20 000 in 47.84% of the cases and in the range of CNY 20 000 to 50 000 in 26.35%.

### Multivariate logistic regression analysis of independent influencing factors affecting the judgment results of the included second-instance and retrial cases

According to the correlation analysis, we initially identified several variables related to the judgment results of the included second-instance and retrial cases. To further explore the risk factors that may influence these outcomes, we determined meaningful variables by combining correlation analysis results with our relevant work experience. Appeal originator, outcomes of second-instance and retrial cases, judicial identification status, violations of medical treatment and nursing routines, and non-standard medical document writing were independent variables, and the judgment results were dependent variables; a stepwise regression method was used to construct the unconditional multivariate logistic regression model.

As shown in Table 2, appeal originator [patient side: OR = 18.809 (95% CI 11.854–29.845); both sides: OR = 22.168 (95% CI 12.249–40.117)], change of the original verdict (OR = 5.936, 95% CI 3.875–9.095), judicial identification (OR = 6.395, 95% CI 4.818–8.487), violations of medical treatment and nursing routines (OR = 8.783, 95% CI 6.658–11.588), and non-standard medical document writing (OR = 8.500, 95% CI 4.805–15.037) were independent risk factors for medical personnel to lose a lawsuit, which with a statistically significant difference.

**Table 2:** Multivariate logistic regression analysis of independent influencing factors affecting the judgment results of the included second-instance and retrial cases

Variables	B	S.E	Wals	*P* value	OR	95% CI
Appeal originator						
Medical side			240.059		1.000	
Patient side	2.934	0.236	155.174	< 0.001	18.809	(11.854, 29.845)
Both parties	3.099	0.303	104.828	< 0.001	22.168	(12.249, 40.117)
Outcomes of second-instance and retrial cases						
Affirmed the original judgement					1.000	
Changed the original verdict	1.781	0.218	66.946	< 0.001	5.936	(3.875, 9.095)
Judicial identification status						
Without identification					1.000	
With identification	1.855	0.144	165.025	< 0.001	6.395	(4.818, 8.487)
Violations of medical treatment and nursing routines						
Not violated					1.000	
Violated	2.173	0.141	236.202	< 0.001	8.783	(6.658, 11.588)
Medical records						
Standard					1.000	
Non-standard	2.140	0.291	54.076	< 0.001	8.500	(4.805, 15.037)

## Discussion

We conducted a multi-dimensional analysis of the judicial decisions of 3172 second-instance and retrial cases among all medical damage liability disputes in China during 2018. Our study clarified the characteristics of the second-instance and retrial cases and determined the key important points for preventing medical disputes. Furthermore, we showed that the patient as appeal originator, both the patient and medical side as appeal originator, changes in the original verdict, judicial identification, violations of medical treatment and nursing routines, and non-standard medical document writing were independent risk factors for medical personnel losing a lawsuit. Similarly, it was reported that physicians’ negligence in diagnosis and post-operation management was 30.8% and 27.7%, respectively, by analysis of lawsuit cases in the department of surgery in Korea [31]. Our study could help medical staff to improve their awareness and ability to prevent medical disputes and provides a basis for medical management departments to develop scientific and reasonable medical dispute avoidance strategies, ultimately promoting the continuous improvement of medical quality. Based on the analysis of the relevant results of this study, several noteworthy viewpoints are summarized below.

### Medical institutions with clear responsibilities should actively seek mediation solutions in the first instance through the court’s mediation mechanism to end disputes as soon as possible and avoid higher litigation costs

Because there is always an “expectations gap” between expectation and outcome from the patient side, the possibility of stopping judgment in the first instance is small [32–34]. The results of our study showed that 48.04% of second-instance and retrial cases were unilateral appeals by the patient. As the initiator of the medical damage dispute lawsuit and the main party to file the appeal, the patient often strives to maximize litigation interests through the second-instance and retrial cases procedure. Therefore, under the premise of clear responsibility, medical institutions could actively settle cases through first-instance mediation, which can reduce litigation costs and save time. This suggestion is consistent with previous studies [35, 36].

### Both the medical side and the patient side should pay attention to the medical expert testimony procedure in the first instance, especially in the initial medical authentication

In the case of medical damage liability disputes, judges generally do not have a medical professional knowledge background and often need to rely on judicial identification conclusions in accordance with the law to make judgments. Therefore, the medical expert testimony procedure is particularly important in medical damage liability disputes. Our statistical data showed that 95% of the appraisal opinions determined the direction of the judgment of these cases. Moreover, once the judicial identification conclusions are made, and there is no apparent procedural violation, the opportunity to apply for re-appraisal is minimal, accounting for only 8.71% of the cases in our study. Therefore, the medical and patient sides should pay attention to the medical expert testimony procedure in the first instance, especially in the initial medical authentication.

### Judicial administration should provide more legal aid resources to the patient side, better safeguard the rights of the patients, resolve the contradiction between the medical side and the patient side

Medical damage liability disputes involve the fields of law and medicine and are highly professional. In the context of epistemic injustice, the patient is frequently considered inferior and lacks support [34]. In our study, the proportion of patients entrusting lawyers was 9.24% lower than among medical appellants (21.75%); however, the total proportion of patients appealing was higher than the number of doctors, indicating a potential correlation between the high proportion of patients appealing and the low proportion of hiring lawyers. Therefore, in the litigation process, judicial administration should provide more legal advice or help to patients, acting in the patients’ best interests [10].

### Medical institutions should reduce the occurrence of medical damage liability disputes by constructing medical quality control systems and formulating medical risk prevention strategies and early warning mechanisms

Due to the hierarchical diagnosis and treatment system in China, the first hospitals visited are generally county- and district-level institutions. Most of the patients referred to municipal or provincial hospitals are critically ill; thus, there is a greater possibility of medical disputes. Therefore, in municipal and provincial hospitals, medical quality control, risk prevention, and early warning mechanisms should be strengthened when treating critically ill patients across the course of service, especially when a patient death occurs for no apparent reason or patients are dissatisfied with the treatment outcomes. This finding is consistent with previous studies [10, 28, 29].

Moreover, previous studies have evaluated the incident reporting system for clinical risk management and suggested that a correct use of the incident reporting system would lead to a reduction in litigation between the medical and the patient sides [37]. In addition, a medical risk prevention and early warning model established as part of the hospital management information system could help to institute early detection and intervention and prevent medical disputes [38].

### Medical institutions should cultivate medical staff’s consciousness of legal practice and strengthen medical staff’s training in practicing according to the law

Medical risks are accompanied by medical activities. The particularity of the medical profession requires medical personnel to develop good habits of practicing according to law, rules, and humanization. Poor investment in training for medical personnel can lead to medical errors and disputes [39]. Medical institutions must constantly cultivate the concept of medical staff practicing according to law, establish a standardized training system for the medical staff on admission, and continuously improve medical and doctor–patient communication skills [10, 23].

Over half of the patients question the authenticity of medical documents in the litigation process [30]. Medical staff should be educated and trained on the standardization of medical document writing and medical notification to prevent defects in medical document writing and improper medical notification, which lead to medical-side compensation or indemnification responsibility, although no responsibility could be determined after the judicial identification. Additionally, some doctors may forge or change medical documents to cover up their possible irregularities to reduce the risk of medical disputes [30]. Hence, patients should actively seal and copy medical documents in medical disputes to ensure the evidence is retained and to safeguard their rights and interests.

### Medical institutions should strengthen medical risk prevention education for medical and nursing staff in surgery, obstetrics and gynecology, and internal medicine departments

In this study, surgery, obstetrics and gynecology, and internal medicine departments are the top three departments that are involved in the second-instance and retrial cases. The number of cases involving surgery departments was the largest, accounting for 38.24% of the cases. Obstetrics and gynecology departments accounted for 16.65%. Internal medicine departments accounted for 16.24%. Moreover, outpatient and emergency, and pediatrics, and intensive care unit departments are also likely to face medical disputes. These results are consistent with previous studies [40, 41]. Thus, all medical institutions should strengthen medical risk prevention education for medical and nursing staff in these departments. In addition to paying attention to the duty of care and strictly complying with medical care routines, medical staff should also pay attention to notifications of surgical plans and surgical risk and the standardization of surgical records. Moreover, medical staff should follow guidelines to protect themselves from medical disputes [42].

## Research limitations

Due to the research approach, this study focused on medical disputes concluding in litigation; the medical disputes solved through reconciliation and mediation by the health administrative department are not discussed. In addition, in the provincial spatial scale correlation analysis, the spatial scale is too large, and the sample size is relatively small; future studies are encouraged to update this study and confirm the robustness of the results.

## Conclusion

Our study clarified the characteristics of second-instance and retrial cases among all medical damage liability disputes in China from multiple perspectives and identified the independent risk factors for medical personnel losing a lawsuit. This study could help medical institutions prevent and reduce medical disputes.

## Supplementary Information

**Additional file 1: Table S1.** Variables analysis and value assignment.

## Data Availability

The datasets generated and analyzed during the current study are available in the China Judgment Online System repository, http://wenshu.court.gov.cn/.

## References

[1] Wyatt R, Anderson-Drevs K, Van Male LM (2016). Workplace violence in health care: a critical issue with a promising solution. JAMA.

[2] Phillips JP (2016). Workplace violence against health care workers in the United States. N Engl J Med.

[3] Civilotti C, Berlanda S, Iozzino L (2021). Hospital-based healthcare workers victims of workplace violence in Italy: a scoping review. Int J Environ Res Public Health.

[4] WHO (2019). Violence against health workers.

[5] Liu J, Gan Y, Jiang H, Li L, Dwyer R, Lu K, Yan S, Sampson O, Xu H, Wang C, Zhu Y, Chang Y, Yang Y, Yang T, Chen Y, Song F, Lu Z (2019). Prevalence of workplace violence against healthcare workers: a systematic review and meta-analysis. Occup Environ Med.

[6] Varghese A, Joseph J, Vijay VR, Khakha DC, Dhandapani M, Gigini G, Kaimal R (2022). Prevalence and determinants of workplace violence among nurses in the South-East Asian and Western Pacific Regions: a systematic review and meta-analysis. J Clin Nurs.

[7] Jaradat Y, Nielsen MB, Kristensen P, Nijem K, Bjertness E, Stigum H, Bast-Pettersen R (2016). Workplace aggression, psychological distress, and job satisfaction among Palestinian nurses: a cross-sectional study. Appl Nurs Res.

[8] Jaradat Y, Birkeland Nielsen M, Kristensen P, Bast-Pettersen R (2018). Job satisfaction and mental health of Palestinian nurses with shift work: a cross-sectional study. Lancet.

[9] Zhao S, Xie F, Wang J, Shi Y, Zhang S, Han X, Sun Z, Shi L, Li Z, Mu H, Liu X, Liu W, Gao L, Sun T, Fan L (2018). Prevalence of workplace violence against Chinese nurses and its association with mental health: a cross-sectional survey. Arch Psychiatr Nurs.

[10] Ma Y, Wang L, Wang Y, Li Z, Zhang Y, Fan L, Ni X (2021). Causes of hospital violence, characteristics of perpetrators, and prevention and control measures: a case analysis of 341 serious hospital violence incidents in China. Front Public Health.

[11] Liang F, Hu S, Guo Y (2022). The association between fear of malpractice and burnout among Chinese medical workers: the mediating role of legal consciousness. BMC Psychiatry.

[12] Guo Y, Hu S, Liang F (2021). The prevalence and stressors of job burnout among medical staff in Liaoning, China: a cross-section study. BMC Public Health.

[13] Chen S, Lin S, Ruan Q, Li H, Wu S (2016). Workplace violence and its effect on burnout and turnover attempt among Chinese medical staff. Arch Environ Occup Health.

[14] Ma Y, Wang Y, Shi Y, Shi L, Wang L, Li Z, Li G, Zhang Y, Fan L, Ni X (2021). Mediating role of coping styles on anxiety in healthcare workers victim of violence: a cross-sectional survey in China hospitals. BMJ Open.

[15] Fang H, Zhao X, Yang H, Sun P, Li Y, Jiang K, Li P, Jiao M, Liu M, Qiao H, Wu Q (2018). Depressive symptoms and workplace-violence-related risk factors among otorhinolaryngology nurses and physicians in Northern China: a cross-sectional study. BMJ Open.

[16] Li X, Wu H (2021). Does psychological capital mediate between workplace violence and depressive symptoms among doctors and nurses in Chinese general hospitals?. Psychol Res Behav Manag.

[17] Liu J, Zheng J, Liu K, Liu X, Wu Y, Wang J, You L (2019). Workplace violence against nurses, job satisfaction, burnout, and patient safety in Chinese hospitals. Nurs Outlook.

[18] Studdert DM, Mello MM, Sage WM, DesRoches CM, Peugh J, Zapert K, Brennan TA (2005). Defensive medicine among high-risk specialist physicians in a volatile malpractice environment. JAMA.

[19] He AJ (1982). The doctor–patient relationship, defensive medicine and overprescription in Chinese public hospitals: evidence from a cross-sectional survey in Shenzhen city. Soc Sci Med.

[20] Kakemam E, Arab-Zozani M, Raeissi P, Albelbeisi AH (2022). The occurrence, types, reasons, and mitigation strategies of defensive medicine among physicians: a scoping review. BMC Health Serv Res.

[21] Survey report of physicians in 2021. 2021. https://www.soc.tsinghua.edu.cn/info/1197/1411.htm. Accessed 26 July 2022.

[22] Survey report of physicians in 2021. 2021. https://www.dxy.cn/bbs/newweb/pc/post/44950677. Accessed 26 July 2022.

[23] Mata ÁNS, de Azevedo KPM, Braga LP, de Medeiros G, de Oliveira Segundo VH, Bezerra INM, Pimenta I, Nicolás IM, Piuvezam G (2021). Training in communication skills for self-efficacy of health professionals: a systematic review. Hum Resour Health.

[24] Jiang MM, Wu ZY, Tu AX (2023). Research on the cooperative governance path of multiple stakeholders in doctor–patient disputes under the environment of information asymmetry. Int J Environ Res Public Health.

[25] Yin T, Liu Z, Xu Y (2019). Analysis of crisis management of medical disputes in China and Australia: a narrative review article. Iran J Public Health.

[26] Dossett ML, Kohatsu W, Nunley W, Mehta D, Davis RB, Phillips RS, Yeh G (2013). A medical student elective promoting humanism, communication skills, complementary and alternative medicine and physician self-care: an evaluation of the HEART program. Explore.

[27] Montgomery L, Loue S, Stange KC (2017). Linking the heart and the head: humanism and professionalism in medical education and practice. Fam Med.

[28] Li N, Wang Z, Dear K (2019). Violence against health professionals and facilities in China: evidence from criminal litigation records. J Forensic Leg Med.

[29] Cai R, Tang J, Deng C, Lv G, Xu X, Sylvia S, Pan J (2019). Violence against health care workers in China, 2013–2016: evidence from the national judgment documents. Hum Resour Health.

[30] Li H, Wu X, Sun T, Li L, Zhao X, Liu X, Gao L, Sun Q, Zhang Z, Fan L (2014). Claims, liabilities, injures and compensation payments of medical malpractice litigation cases in China from 1998 to 2011. BMC Health Serv Res.

[31] Jung JY, Kim SY, Kim DG, Kim CB, Chi KC, Kang WK, Lee W (2018). Analysis of lawsuit cases in the Department of Surgery in Korea. Ann Surg Treat Res.

[32] Bismark MM, Spittal MJ, Gogos AJ, Gruen RL, Studdert DM (2011). Remedies sought and obtained in healthcare complaints. BMJ Qual Saf.

[33] Friele RD, Reitsma PM, de Jong JD (2015). Complaint handling in healthcare: expectation gaps between physicians and the public; results of a survey study. BMC Res Notes.

[34] Dijkstra RI, Elbers NA, Friele RD, Pemberton A (2022). Medical Dispute Committees in the Netherlands: a qualitative study of patient expectations and experiences. BMC Health Serv Res.

[35] Busby G (2015). Incidence and cost of workplace violence. J Emerg Nurs.

[36] Speroni KG, Fitch T, Dawson E, Dugan L, Atherton M (2014). Incidence and cost of nurse workplace violence perpetrated by hospital patients or patient visitors. J Emerg Nurs.

[37] Ferorelli D, Solarino B, Trotta S, Mandarelli G, Tattoli L, Stefanizzi P, Bianchi FP, Tafuri S, Zotti F, Dell'Erba A (2020). Incident reporting system in an Italian University Hospital: a new tool for improving patient safety. Int J Environ Res Public Health.

[38] Xu P, Fan Z, Li T, Wang L, Sun Q, Du X, Lian B, Zhang L (2015). Preventing surgical disputes through early detection and intervention: a case control study in China. BMC Health Serv Res.

[39] Ending violence against doctors in China. Lancet. 2012;379:1764. 10.1016/S0140-6736(12)60729-6 22579308

[40] Baker KA (2019). Violence in the gastroenterology setting. Gastroenterol Nurs.

[41] Wang M, Liu GG, Zhao H, Butt T, Yang M, Cui Y (2020). The role of mediation in solving medical disputes in China. BMC Health Serv Res.

[42] Choi S, Shin S, Lee W, Choi SM, Kang SW (2020). Medicolegal lessons learned from thyroidectomy-related lawsuits: an analysis of judicial precedents in South Korea from 1998 to 2019. Gland Surg.

