# Supplementary material for: Analysis of the characteristics and risk factors affecting the judgment results of medical damage liability disputes in 3172 second-instance and retrial cases in China
Source: Hum Resour Health. 2023 Jun 29;21:53. doi: 10.1186/s12960-023-00832-6 (PMC10308770; doi:10.1186/s12960-023-00832-6)
Supplement: Supplementary file 1 — Additional file 1: Table S1. Variables analysis and value assignment. [file 12960_2023_832_MOESM1_ESM.docx]

**Table S1. Variables analysis and value assignment**

| **Variables** | **Value assignment** |
| --- | --- |
| Judgment results | Medical side wins = 0; Medical side loses = 1 |
| Appeal originator | Medical side = 0; Both sides = 1; Patient side = 2 |
| Level of the trial court | Intermediate court = 0; Superior court = 1 |
| Procurement of attorney | Without entrusted = 0; Entrusted by the medical side = 1;  Entrusted by the patient side = 2; Entrusted by both parties = 3 |
| Outcomes of second-instance and retrial cases | Affirmed the original judgement = 0;  Changed the original verdict = 1 |
| Hospital category | Township hospital = 0; County and district hospital = 1; Municipal hospital = 2; Private hospital and infirmary = 3; Enterprise-owned hospital = 4; Military hospital = 5; Provincial hospital = 6 |
| Number of defendants | One medical institution = 0; Two medical institutions = 1;  Three medical institution = 2 |
| Departments in dispute | Outpatient and emergency = 0; Pediatrics = 1; Obstetrics and gynecology = 2; Intensive care unit = 3; Internal medicine = 4; Surgery = 5; Other departments = 6 |
| Judicial identification status and proportion of responsibilities | No identification = 0; No responsibility = 1; Minor responsibility = 2; Secondary responsibility = 3; Equal responsibility = 4;  Main responsibility = 5; Full responsibility = 6 |
| Adoption of appraisal opinions | Fully adopted = 1; Partially adopted and aggravation = 2;  Partially adopted and alleviation = 3; Completely rejected = 4 |
| Location of judicial identification | Appraisal institutions in the province = 0;  Appraisal institutions outside the province = 1 |
| Re-identification status | No re-identification = 0; Illegal identification procedures = 1; Insufficiency of evidence = 2; Testimony others = 3; Lack of accreditation = 4 |
| Comparison of re-identification and initial judicial identification | Consistency = 0; Inconsistency = 1 |
| Appraiser appearance in court | Appraisers do not appear in court = 0; Appraisers appear in court and the results are in agreements = 1; Appraisers appear in court and the results are aggravated = 2; Appraisers appear in court and the results are alleviated = 3 |
| Amount of compensation (CNY) | No compensation = 0; 0-100,000 = 1; 100,000-500,000 = 2;  500,000-1,000,000 = 3; More than 1,000,000 = 4 |
| Mental damage compensation (CNY) | No compensation = 0; 0-20,000 = 1; 20,000-50,000 = 2;  50,000-100,000 = 3; More than 100,000 = 4 |
| Informed consent notification | Not violated = 0; Violated = 1 |
| Medical records | Standard = 0; Non-standard = 1 |
| Violations of medical treatment and nursing routines | Not violated = 0; Violated = 1 |
